# Effect of Exogenous General Plant Growth Regulators on the Growth of the Duckweed *Lemna minor*

**DOI:** 10.3389/fchem.2018.00251

**Published:** 2018-07-09

**Authors:** Desi Utami, Ami Kawahata, Masayuki Sugawara, Rahul N. Jog, Kyoko Miwa, Masaaki Morikawa

**Affiliations:** Graduate School of Environmental Science, Hokkaido University, Sapporo, Japan

**Keywords:** *Lemna minor*, plant growth regulators, gibberellic acid, indole-3-acetic acid, salicylic acid, 1-aminocyclopropane-1-carboxylic acid, aminoethoxyvinylglycine, ascorbic acid

## Abstract

Gibberellic acid (GA_3_), indole-3-acetic acid (IAA), salicylic acid (SA), abscidic acid (ABA), jasmonic acid (JA) 1-amino cyclopropane-1-carboxylic acid (ACC) and aminoethoxyvinylglycine (AVG) are popular growth regulators of plants. However, the effects of their exogenous addition on the biomass production of aquatic plants, including *Lemnoideae* plants, “duckweeds,” are largely unknown. In this study, the growth of *Lemna minor* was tested for 10 d in Hoagland medium containing each compound at different concentrations of 0–50 μM. GA_3_, IAA, and SA were found to have no apparent positive effect on the growth at all concentrations tested. Conversely, ACC and JA moderately and AVG and ABA severely inhibited the growth of *L. minor*. Among the tested compounds, ascorbic acid had an apparent growth-promoting effect.

## Introduction

Duckweeds, a general name for plants belonging to the *Lemnoideae* subfamily, represent the smallest free-floating monocotyledonous aquatic plants with vascular systems. *Lemnoideae* includes five genera: *Landoltia, Lemna, Spirodela, Wolffiella*, and *Wolffia*. The plant body consists of “frond,” leaf-like structure, and “root” (except for *Wolffiella* and *Wolffia*, which are rootless duckweeds). Some duckweeds develop seed-like “turion,” which contains high starch and anthrocyanin depending on the temperature, light and nutrient conditions (Smart and Trewavas, 1983). Although duckweeds are flowering plants, they primarily reproduce asexually by vegetative propagation. Duckweeds are robust, environmentally well-adapted, and ubiquitously distributed from 55° S to 70° N. They were recently highlighted as a potential biomass.

The following are the advantages of duckweeds as biomass: (1) they grow at significantly high rates; (2) they grow on wastewater and uptake and remove contaminant minerals as nutrients; (3) they can be used as slow, but low-cost wastewater treatment systems driven by sun light; (4) they provide nonfood competitive biomass with high protein and starch, but low lignin and cellulose content; and (5) they change their constituent starch and protein content depending on the growth condition. Thus, duckweeds are expected to be one of the next-generation biomass resources to be associated with animal feed, biofuel and starch-based green chemistry (Cheng, 2010; Baliban et al., 2013). Increase in the growth rate of duckweeds should contribute to the increasing availability of duckweed biomass and allow the prevention of global warming and realization of sustainable industries and societies. Increasing attention is being paid to producing duckweeds coupled with wastewater treatment (Xu et al., 2012; Zhao et al., 2014; Toyama et al., 2018).

Researchers have long attempted to increase the growth of plants by adding chemical compounds in addition to mineral fertilizers. In 1917, Bottomley reported that water extracts from bacterized peat contained certain growth-promoting organic compounds for *Lemna minor*, namely, auximones (Bottomley, 1917, 1920). Gorham clearly showed that sucrose at concentrations higher than 1% increased the growth of *Spirodela polyrhiza* (Gorham, 1945). Landolt found that sucrose promoted the growth of all *Lemnoideae* strains tested under low light intensities, and this effect was attenuated under higher intensities (Landolt, 1957). Lactic acid has been known to act as a plant growth regulator for carrot, marigold, sunflower and sugarcane (Hildebrandt et al., 1954). Further, dimers or larger polymers of l-lactic acid at 1,000 ppm were shown to promote the growth of both *L. minor* and *Zea may*s L. by 2-fold (Kinnersley et al., 1990). Organic fertilizer is now widely used in crop and vegetable farms.

Developing technologies that enable duckweed growth by the addition of chemical compounds requires the understanding of the basic traits of duckweeds exposed to exogenously added representative bioactive organic compounds. Plant growth regulator compounds, including plant hormones, profoundly influence the growth and differentiation of plant cells, tissues, and organs. Many biotic and abiotic chemical compounds are known to affect plants; for example, regulation of cell size and cell division by ethylene, regulation of cell cycle by auxins (IAA) and cytokines, induction of seed germination and stem elongation by gibberellins (GA), maintenance of seed dormancy by abscisic acid (ABA) and an endogenous signaling against pathogens and abiotic stresses by salicylic acid (SA) and jasmonic acid (JA).

In aquatic plants such as *L. minor*, the effects of growth regulators on biomass production have rarely been investigated. Gibberellic acid (GA_3_) has been reported to homeopathically potentize and reduce the growth rate of *L. gibba* (Scherr et al., 2009). However, the reproducibility of the result was confirmed later by the same group, showing that growth was increased by GA_3_ (Majewsky et al., 2014). Scherr et al. (2007) also found that eleven potentized compounds, including IAA, decreased the growth of *L. gibba*. Idris et al. (2007) prepared three mutants of plant growth-promoting *Bacillus amyloliquefaciens* FZB42 that showed reduction of 15–38% in the level of IAA. The growth-promoting activity of these mutants against *L. minor* concomitantly reduced to 17–19%, suggesting that IAA is a predominant plant growth-promoting factor. These controversial and confusing findings prompted us to determine comprehensively the effect of exogenous general plant growth regulator compounds on the growth of *L. minor*.

## Materials and methods

### Duckweed culture

Sterile *L. minor* RDSC #5512 isolated from the Botanical Garden of Hokkaido University was used in this study. About 50 fronds of *L. minor* were aseptically transferred every 10 d to new 500 mL flasks containing 250 mL Hoagland medium for subculture. Hoagland medium contains per liter of 36.1 mg KNO_3_, 293 mg K_2_SO_4_, 103 mg MgSO_4_-7H_2_O, 147 mg CaCl_2_-2H_2_O, 5 mg NaHPO_4_-2H_2_O, 0.95 mg H_3_BO_3_, 0.39 mg MnCl_2_-4H_2_O, 0.03 mg CuSO_4_-5H_2_O, 0.08 mg ZnSO_4_-7H_2_O, 0.23 mg H_2_MoO_4_, and 3.33 mg FeSO_4_-7H_2_O. The pH was adjusted to 7.0 using sodium hydroxide. Plant culture conditions were 28°C, 60–70% humidity, 5000 lux (75 μmol/m^2^/s photon density) and photoperiod of 16 h light / 8 h dark. Sterility of *L. minor* was confirmed when no bacterial colony was formed on R2A agar plate after incubation at 30°C for 3 d. R2A agar contained per liter 0.50 g each of yeast extract, proteose peptone (Difco no. 3), casamino acids, glucose, and soluble starch; 0.3 g each of Na-pyruvate and K_2_HPO_4_; and 0.05 g MgSO_4_-7 H_2_O. The pH was adjusted to 7.2 using sodium hydroxide. Agar was added at 15 g before autoclaving.

### Plant growth regulators

The plant growth regulator compounds used in this study were GA_3_ (Sigma Aldrich), indole-3-acetic acid (IAA; Wako Pure Chemical Ind. Ltd.), SA (Wako), 1-aminocyclopropane-1-carboxylic acid (ACC; Wako), aminoethoxyvinylglycine (AVG; Sigma Aldrich). Ascorbic acid (Wako) was also tested for the plant growth experiment. Stock solution of chemicals was prepared in MilliQ water and filter-sterilized.

### Evaluation of the growth of *L. minor*

Sterile *L. minor* was transferred to Petri dish containing appropriate amount of Hoagland medium, its two fronds were separated using a pair of forceps and inoculated into 100 mL flasks containing each compound in 50 mL Hoagland medium. Flasks were prepared in three replicates for every experiment. The effects of these compounds on plant growth were quantified by evaluating four parameters: frond numbers, fresh weight or dry weight, root length and chlorophyll *a*+*b* content. After the number of fronds was counted, five plants of 10 d culture were randomly obtained from each flask, and the length of the longest roots was measured as the root length for every treatment. For the fresh weight, whole plants in the flask were carefully removed, blotted dry on paper towel, and weighed by a micro-scale (Sartorius, Göttingen Germany). Dry weight was measured in nylon tea bag filters after freeze drying (FDU-1110; EYELA).

Chlorophyll contents of *L. minor* were measured as previously described (Suzuki et al., 2014). Briefly, several plants were placed into 2 mL Eppendorf tubes containing 0.5 g of 0.1 mm glass beads (YGB01, Yasui Kikai, Japan). Next, 1 mL of cold ethanol previously saturated with Ca(CO_3_)_2_ was added and vigorously shaken by using a Multi-beads shocker MB755U(S) (Yasui Kikai) at 2,700 vibration per minute for 300 s at 4°C. After glass beads and cell debris were removed by centrifugation, the chlorophyll content was quantified by measuring photometric absorption at 649 and 665 nm. The concentration in units of μg/mL of chlorophyll *a* was calculated as [13.5275 (A_665_) – 5.2007 (A_649_)] and chlorophyll *b* as [−7.0741 (A_665_) + 22.4327 (A_649_)]. The chlorophyll content was determined as mg chlorophyll *a*+*b*/g fresh weight of specimens. Statistical significance of each value was validated by unpaired t-test and one-way ANOVA using triplicate samples.

## Results and discussion

### GA_3_

Rice farmers of Japan had long noted a fungal disease called “foolish seedling” or *bakanae* disease that causes rice plants to grow taller and reduces seed production. Plant pathologists found that this symptom in rice plants was caused by chemicals secreted by a pathogenic fungus, *Gibberella fujikuroi*. These fungal compounds possessing plant growth promoting activity were later named gibberellins A_1_, A_2_, and A_3_ or gibberellic acid (Takahashi et al., 1955). GA_3_ can also promote the growth of maize by inhibiting peroxidase secretion into the apoplast (Fry, 1979). The treatment of maize plants with GA_3_ increased the length of the leaf blade elongation zone compared with that in the control via an increase in cell division and cell elongation duration (de Souza and MacAdam, 2001). In other plants such as potatoes as well, GA_3_ treatments significantly increased the number of sprouts, stems, and tubers at very high concentrations of 100 mM in the cultivar “Fambo,” but the total biomass did not increase (Virtanen et al., 2013). Oota and Tsudzuki (1971) reported that the number of fronds was increased to 125% by the application of 100 μM GA_3_. Inada and Shimmen (2000) investigated the effects of the exogenous addition of GA_3_ to *L. minor*. These researchers observed no significant changes in root length up to 1 μM, but found that it slight decrease at 10 μM concentration. Conversely, they also reported that exogenous addition of >1 mM GA_3_ to the root segment of *L. minor* for 12 h inhibited root elongation in a concentration-dependent manner up to 1 μM (Inada and Shimmen, 2000). These researchers attributed this inconsistency to the fact that the root tip part was not included in the root segments used and the duration of exposure to GA_3_ differed. However, they reported that uniconazole-P (Un-P), a gibberellin biosynthesis inhibitor, also inhibited the elongation of segments at 10 nM similar to the decrease in the root length in the experiment involving whole plants. These data show that the effect of GA_3_ on the growth of *L. minor* is still controversial.

**Figure 1:**
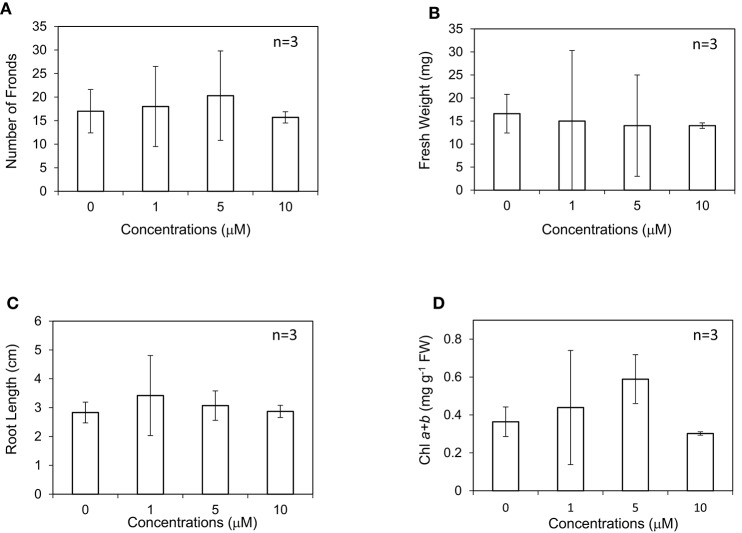
Effect of gibberellic acid (GA_3_) on the frond number **(A)**, fresh weight **(B)**, root length **(C)**, and chlorophyll content **(D)** of *Lemna minor*. Each value was measured after 10 d of cultivation. SD values are shown in line segments.

In our experiment, *L. minor* was grown in the presence of different concentrations of GA_3_ ranging from 0 to 50 μM in Hoagland medium (Figure 1). After 10 d, the growth traits of *L. minor*, such as the frond number, root length, fresh weight, and chlorophyll content, were observed. Both the number of fronds and root length were not considerably different among the treatments, suggesting that *L. minor* is insensitive to exogenous GA_3_ at these concentrations (Figures 1A,C). This result was also supported by the fresh weight of the plants (Figure 1B). Conversely, the chlorophyll content was increased from 0.37 to 0.59 mg g^−1^ fresh weight after treatment with 5 μM GA_3_ (Figure 1D). On the other hand, we observed that ABA, another endonegenous stress signaling compound and an antagonist of GA severely inhibited the growth of *L. minor* (Supplementary Figure 1).

### IAA

Indole-3-acetic acid is the principal auxin in plants; it controls many important physiological processes, including cell enlargement and division, tissue differentiation, and responses to light and gravity (Taiz and Zeiger, 1998). Auxins stimulate cell elongation and influence a host of other developmental responses such as root initiation, vascular differentiation, tropic responses, apical dominance, and the development of auxiliary buds, flowers and fruits. Auxins are synthesized in the stem and root apices and transported through the plant axis. Several other indole derivatives, as precursors to IAA, are known to express auxin activity, probably by converting to IAA in the tissue. This hormone is widely used in agriculture and horticulture to prevent leaf abscission and fruit drop, promote flowering and fruiting, and control weeds. Exogenous application of IAA has been reported to increase the growth of the root and shoot of wheat seedlings and to protect plants against stress (Egamberdieva, 2009). Yang et al. (1993) reported significant promotion of stem elongation in pea seedlings by exogenous IAA at concentrations from 50 to 1,000 μM. Conversely, exogenous IAA at 1 μM was found to retard germination and decrease the fresh weight of germinated soybean seeds. A small, but significant growth promotion by IAA up to 25 ppm (143 μM) was reported for crude culture of *L. minor* (Blackman and Robertson-Cuninghame, 1954); they also noted frond epinasty and root inhibition at higher concentrations. Conversely, Inada and Shimmen (2000) reported that exogenous IAA up to 1 μM inhibited the elongation of root segments in *L. minor*.

**Figure 2:**
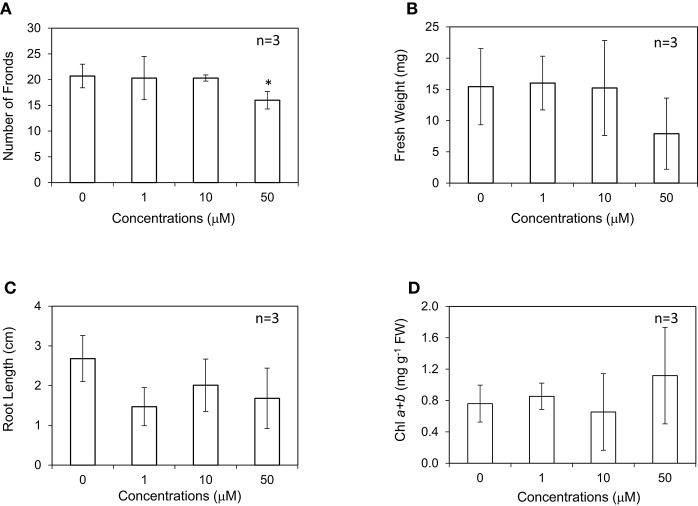
Effect of indole-3-acetic acid (IAA) on the frond number **(A)**, fresh weight **(B)**, root length **(C)**, and chlorophyll content **(D)** of *Lemna minor*. Each value was measured after 10 d of cultivation. SD values are shown in line segments. *p < 0.05 against 0 μM control.

In this study, *L. minor* was grown in the medium containing different concentrations of IAA at 0–50 μM. The root length was found to decrease by approximately 50% at all the concentrations of IAA higher than 1 μM (Figure 2C; Inada and Shimmen, 2000). Conversely, frond numbers, fresh weight, and chlorophyll content of *L. minor* were not affected by the addition of IAA except for significant growth inhibition at 50 μM (Figures 2A,B,D). Notably, the amount of chlorophyll was not decreased even after treatment with 50 μM IAA. We also tested IAA at 0.1 and 0.5 μM but no plant growth-promoting activity was observed (data not shown). In contrast to the generally positive effects of IAA on the growth of soil plants, no plant growth-promoting activity was observed in our experiment for *L. minor*.

### SA

Salicylic acid is an endogenous signaling compound abundantly produced under abiotic and biotic stresses, including drought, temperature, heavy metals, and pathogen infection. Despite its broad distribution in plants, SA has basal levels differing widely among species, with up to 100-fold differences been reported (Raskin et al., 1990). The role of SA in photosynthetic parameters and short-term acclimation to high light was deduced from the phenotypes shown by *Arabidopsis thaliana* plants with contrasting endogenous SA levels. Moreover, the effects of exogenous SA on photosynthesis parameters were found to differ depending on the dose and plant species tested. High concentrations (1–5 mM) of SA reduced the photosynthetic rate and RuBisCO activity in barley plants (Pancheva and Popova, 1998). *A. thaliana* mutants with different endogenous levels of SA showed growth phenotype that was inversely correlated to SA content (Vicente and Plasencia, 2011).

**Figure 3:**
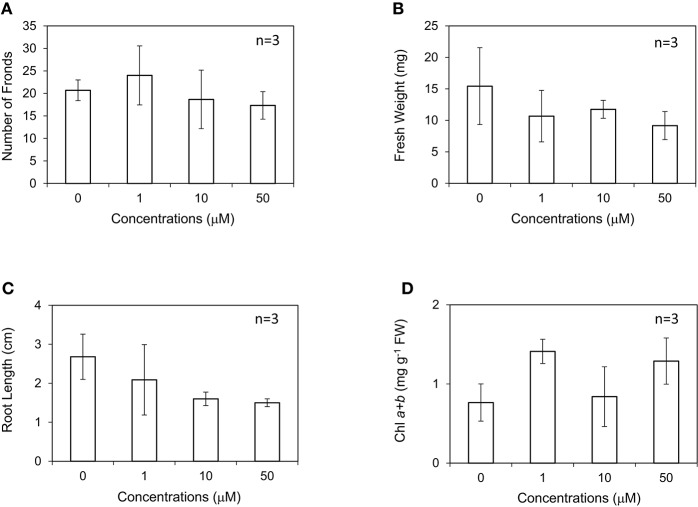
Effect of salicylic acid (SA) on the frond number **(A)**, fresh weight **(B)**, root length **(C)**, and chlorophyll content **(D)** of *Lemna minor*. Each value was measured after 10 d of cultivation. SD values are shown in line segments.

Shimakawa et al. (2012) reported that the amount of endogenous SA was increased under nutrient-poor conditions, and exogenous SA application induced flowering in *Lemna aequinoctialis*. This suggests that SA in *Lemnoideae* plants is also a signaling compound in response to environmental stresses. Recently, the exogenous application of 50 μM SA was reported to reduce the accumulation of Cd, leading to less toxicity (Lu et al., 2018). In this study, *L. minor* was grown in the medium containing different concentrations of SA at 0–50 μM. SA did not significantly affect the number of fronds, but generally and clearly decreased fresh weight and root length of *L. minor* (Figures 3A–C). Although the range of errors is large, the amount of chlorophyll was not found to decrease (Figure 3D). Another endogenous stress signaling compound, JA clearly inhibited the growth of *L. minor* (Supplementary Figure 1).

### ACC

Ethylene affects plant growth at all stages of development from seed germination to organ senescence and promotion of fruit ripening (Corbineau et al., 2004). Ethylene is known to be a key initiator of fast underwater elongation of submerged plants (Jackson, 2008). Treatments of submerged and non-submerged *Oryza sativa* with ethylene stimulated internode elongation (Métraux and Kende, 1983). Moreover, when ethylene synthesis was blocked with aminooxyacetic acid and aminoethoxyvinylglycine (AVG) in partially submerged plants, internode elongation was inhibited. This growth inhibition was reversed when ethylene biosynthesis was restored with ACC, an immediate precursor of ethylene. ABA and ethylene are known to have antagonistic functions for controlling plant growth and development, including seed germination and early seedling development (Cheng et al., 2009). External addition of ACC to plants changes the convergent point between these two signaling pathways. Under light condition, *A. thaliana* shows an elongated hypocotyl and shortened root growth in the presence of 1 μM exogenous ACC (Smalle et al., 1997). When grown in darkness, both the hypocotyls and roots were reduced in the wild type at 0.5 μM and in the *are2* mutant at 10 μM of exogenous ACC (Vanderstraeten and Straeten, 2017).

**Figure 4:**
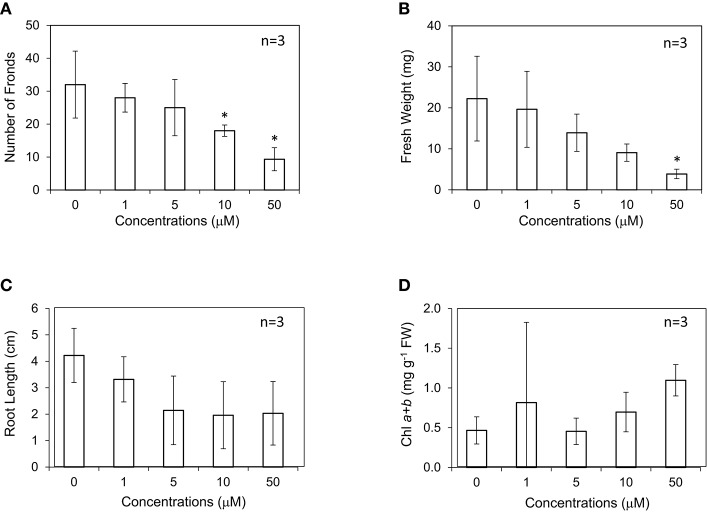
Effect of 1-aminocyclopropane-1-carboxyl acid (ACC) on the frond number **(A)**, fresh weight **(B)**, root length **(C)**, and chlorophyll content **(D)** of *Lemna minor*. Each value was measured after 10 d of cultivation. SD values are shown in line segments. *p < 0.05 against 0 μM control.

Ethylene is known to be a gibbosity regulator of *L. gibba* and *L. minor* (Elzenga et al., 1980); however, its effect on growth has not been investigated. In this study, *L. minor* was grown with different concentrations of ACC under 16-h light photoperiod condition. In contrast to submerged plants, the effects of exogenous ACC on *L. minor* growth were apparently negative (Figures 4A–C). Addition of 1–50 μM of ACC significantly decreased the frond numbers, fresh weight, and root length. This might be explained by the effect of enhanced ethylene production in the plant body that reduced the growth and development. With regard to the effect of ACC on chlorophyll content, although the range of errors was large, the amount of chlorophyll was surprisingly increased up to 50 μM ACC (Figure 4D).

### AVG

AVG is often used as a specific inhibitor of ethylene biosynthesis to analyze the effects of ethylene on plant growth, development and stress response (Abeles et al., 1992) that potentially can mitigate growth suppression under stress condition. Further, the addition of 1 μM of AVG was found to significantly suppress the pistil length of *L. gibba* (Mader, 2004) but no report about the effect of AVG on overall plant growth are yet available.

**Figure 5:**
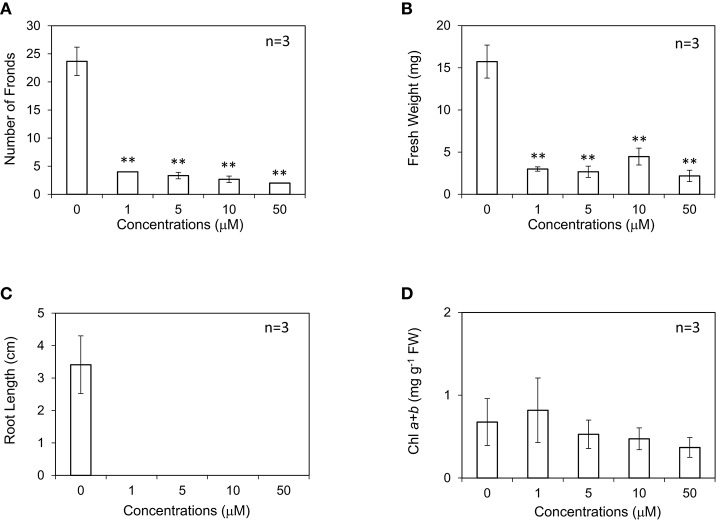
Effect of aminoethoxyvinylglycine (AVG) on the frond number **(A)**, fresh weight **(B)**, root length **(C)**, and chlorophyll content **(D)** of *Lemna minor*. Each value was measured after 10 d of cultivation. SD values are shown in line segments. **p < 0.01 against 0 μM control.

AVG remarkably inhibited the growth of *L. minor*. AVG decreased the frond number, fresh weight and chlorophyll content of *L. minor* at all concentrations (Figures 5A–D). Roots were lost in almost all the surviving plants. Growth of *L. minor* was also impaired by the addition of very low concentration (0.5 μM) of AVG (data not shown). Interestingly, the amount of chlorophyll was decreased, but not as much as the degree of growth defect (Figure 5D). Only this trait can be rationally explained by the inverse effect of ACC and AVG on the biosynthesis of ethylene. However, our experimental results indicate that AVG is not a compound useful for enhanced production of duckweed biomass. Notably, spraying of AVG onto the shoots of cotton blocked ethylene accumulation in the leaves under waterlogging stress. This application subsequently improved leaf growth and enhanced the production of both fruit and cotton cultivars even under non-waterlogging condition (Najeeb et al., 2015).

### AsA

L-Ascorbic acid (AsA) is an abundant water-soluble antioxidant in plants that is capable of scavenging toxic reactive oxygen species (ROS) emitted during photosynthesis. Overproduction of ROS is triggered by various environmental stress-induced oxidative damages. Exogenous AsA is known to protect lipids and proteins against oxidative stresses, thereby promoting the growth of plants. Application of AsA to foliar has been reported to improve the growth of strawberry plants under high temperature condition (44°C; Ergin et al., 2014). For example, exogenous AsA at 3 mM increased cell turgidity of the strawberry plants and hence their growth. Foliar or seed treatment with 20 and 40 mg L^−1^ AsA improved the seedling growth and yield of maize under low temperature stress (Ahmad et al., 2014). Moreover, exogenous application of AsA has been found effective in mitigating the adverse effects of water stress in wheat (Hafez and Gharib, 2016). Once used, AsA can be recycled by several different mechanisms in the plant cells (Gallie, 2013; Akram et al., 2017).

**Figure 6:**
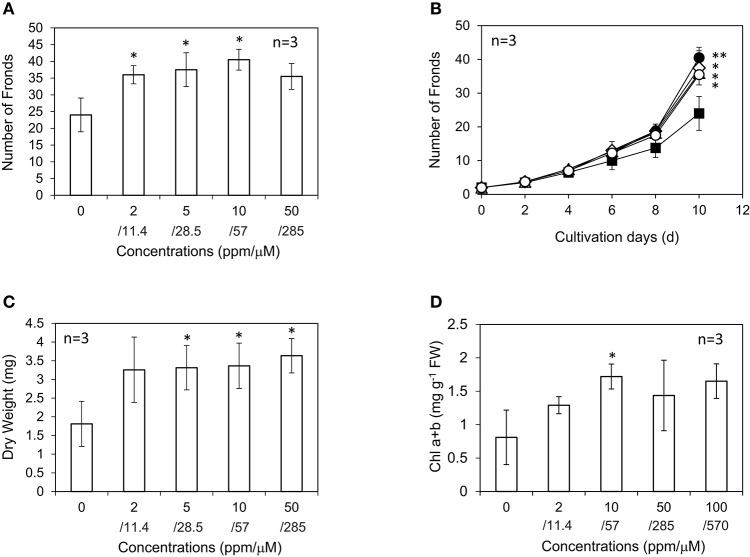
Effect of ascorbic acid on the frond number **(A)**, frond number during 10 d **(B)**, dry weight **(C)**, and chlorophyll content **(D)** of *Lemna minor*. Each value was measured after 10 d of cultivation except for **(B)**. Symbols are 0 ppm, closed square; 2 ppm, open triangle; 5 ppm, open diamond; 10 ppm, closed circle; 50 ppm, open circle. SD values are shown in line segments. *p < 0.05 against 0 μM control. **p < 0.01 against 0 μM control.

AsA showed significant growth-promoting activity in *L. minor*. The highest increase in frond number was observed at 10 ppm, 57 μM AsA, which was 1.7-fold higher than that in the control experiment with no AsA addition (Figures 6A,B). The number of fronds was slightly lower at 50 ppm AsA than at 10 and 5 ppm AsA; however, the dry weight of plants was equivalent or even slightly higher (Figure 6C). All fronds of *L. minor* grown in the presence of AsA showed higher chlorophyll content than that in the control, suggesting that AsA reduced ROS accumulation in the apoplast. The chlorophyll content after treatment with 10 ppm AsA was higher than that in the control (Figure 6D). Recently, Ishizawa et al. (2017) reported that *L. minor* accumulated ROS and expressed an evident antioxidant enzyme activity. Furthermore, the antioxidant enzyme activity was markedly increased in the presence of plant growth-inhibiting bacteria. These results are not inconsistent with our observation that an antioxidant AsA increased the growth of *L. minor*.

## Conclusions

- Exogenous application of the plant hormones gibberellic acid (GA_3_), indole-3-acetic acid (IAA), and salicylic acid (SA) showed no apparent growth-promoting effect on frond number and fresh weight of *L. minor*. Root length was tended to be decreased by IAA and SA. Jasmonic acid (JA) moderately and abscidic acid (ABA) severely inhibited the growth of *L. minor*.
- Growth of *L. minor* was inhibited by both antagonistically functioning compounds in ethylene production, ACC and AVG, where the latter acted more severely. These results suggest that *L. minor*, a *Lemnoideae* plant, behaves uniquely against plant growth regulator compounds.
- AsA significantly promoted the growth of *L. minor* under normal growth condition at concentrations higher than 2 ppm (11.4 μM). To the best of our knowledge, AsA has till date not been shown to promote the growth of *Lemnoideae* plants.

## Author contributions

DU, AK, RJ, and MS designed and performed the experiments and drafted the manuscript. KM and MM interpreted the results revised the manuscript and supervised the study.

### Conflict of interest statement

The authors declare that the research was conducted in the absence of any commercial or financial relationships that could be construed as a potential conflict of interest.
