# Supplementary material for: Effect of Exogenous General Plant Growth Regulators on the Growth of the Duckweed Lemna minor
Source: Front Chem. 2018 Jul 9;6:251. doi: 10.3389/fchem.2018.00251 (PMC6046615; doi:10.3389/fchem.2018.00251)
Supplement: Supplementary file 1 [file Presentation_1.pptx]

## Slide 1
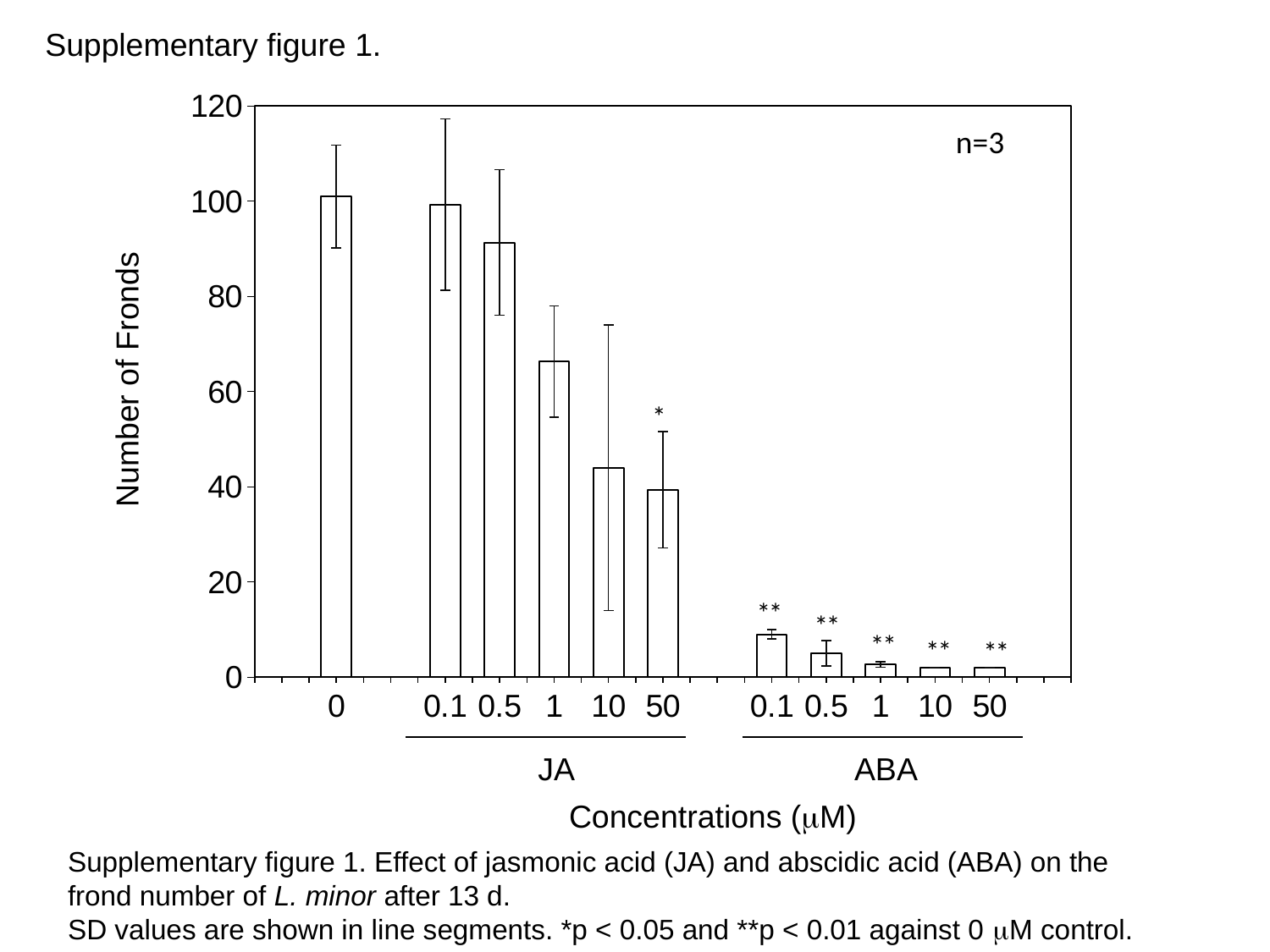

Supplementary figure 1.
### Chart
| Category | |
|---|---|
| | None |
| 0 | 101.0 |
| | None |
| 0.1 | 99.33333333333333 |
| 0.5 | 91.33333333333333 |
| 1 | 66.33333333333333 |
| 10 | 44.0 |
| 50 | 39.333333333333336 |
| | None |
| 0.1 | 9.0 |
| 0.5 | 5.0 |
| 1 | 2.6666666666666665 |
| 10 | 2.0 |
| 50 | 2.0 |
| | None |n=3
Number of Fronds
*
*
*
*
*
*
*
*
*
*
*
JA
ABA
Concentrations (mM)
Supplementary figure 1. Effect of jasmonic acid (JA) and abscidic acid (ABA) on the frond number of L. minor after 13 d.
SD values are shown in line segments. *p < 0.05 and **p < 0.01 against 0 mM control.
